# Mechanisms of progression of chronic kidney disease

**DOI:** 10.1007/s00467-007-0524-0

**Published:** 2007-07-24

**Authors:** Agnes B. Fogo

**Affiliations:** Department of Pathology, Vanderbilt University Medical Center, MCN C3310, Nashville, TN 37232 USA

**Keywords:** Angiotensin, Angiotensin I converting enzyme inhibitors (ACEI), Angiotensin receptors, Angiotensin receptor blockers, Transforming growth factor (TGF)-beta, Glomerulosclerosis, Interstitial fibrosis, Podocytes, Low birth weight

## Abstract

Chronic kidney disease (CKD) occurs in all age groups, including children. Regardless of the underlying cause, CKD is characterized by progressive scarring that ultimately affects all structures of the kidney. The relentless progression of CKD is postulated to result from a self-perpetuating vicious cycle of fibrosis activated after initial injury. We will review possible mechanisms of progressive renal damage, including systemic and glomerular hypertension, various cytokines and growth factors, with special emphasis on the renin–angiotensin–aldosterone system (RAAS), podocyte loss, dyslipidemia and proteinuria. We will also discuss possible specific mechanisms of tubulointerstitial fibrosis that are not dependent on glomerulosclerosis, and possible underlying predispositions for CKD, such as genetic factors and low nephron number.

## Introduction

Chronic kidney disease (CKD) occurs in all age groups, with an incidence in children between 1.5 per million and 3.0 per million. Renal developmental abnormalities (congenital abnormalities of the kidney and urinary tract, CAKUT) are the most common causes of CKD in children. Other diseases commonly underlying CKD in children include focal segmental glomerulosclerosis (FSGS), hemolytic uremic syndrome (HUS), immune complex diseases, and hereditary nephropathies, such as Alport’s disease [1]. The incidence of diabetes, especially type 2, is increasing in children. Although CKD secondary to diabetes usually does not develop until adulthood, early structural lesions of diabetic nephropathy start in childhood [2].

CKD shares a common appearance of glomerulosclerosis, vascular sclerosis and tubulointerstitial fibrosis, suggesting a common final pathway of progressive injury [3]. Adaptive changes in nephrons after initial injury are postulated ultimately to be maladaptive, eventually causing scarring and further nephron loss, thus perpetuating a vicious cycle that results in the end-stage kidney. We will review possible mechanisms of progressive renal damage, which include, but are not limited to, hemodynamic factors, the renin–angiotensin–aldosterone system (RAAS), various cytokines and growth factors, podocyte loss, dyslipidemia, proteinuria, specific mechanisms of tubulointerstitial fibrosis, and possible underlying predispositions for CKD, such as genetic factors and low nephron number.

### Systemic and glomerular hypertension

Systemic hypertension often accompanies renal disease and may both result from, and contribute to, CKD. Progression of CKD is accelerated by hypertension, and control of blood pressure is key in the treatment of CKD. In addition, the glomerulus has a unique structure, with both an afferent and an efferent arteriole, which permits modulation of glomerular perfusion and pressure without corresponding systemic blood pressure change.

The remnant kidney model has been extensively studied to investigate CKD [4]. In this model, one kidney and infarction/removal of two-thirds of the remaining kidney (i.e. five-sixths nephrectomy) results in progressive hyperperfusion, hyperfiltration, hypertrophy and FSGS [4–6]. Additional models with initial podocyte injury, namely the puromycin aminonucleoside and adriamycin models of renal disease, show initial proteinuria and podocyte damage similar to human minimal-change disease, followed by progressive FSGS [7].

Direct micropuncture studies have demonstrated that single nephron function was increased after renal ablation, and led to the hypothesis that hyperfiltration caused sclerosis, setting in motion a vicious cycle of hyperfiltration and glomerulosclerosis [3, 8]. Maneuvers that decreased hyperfiltration, such as low-protein diet, angiotensin I converting enzyme inhibitors (ACEIs), lipid-lowering agents, or heparin, were, indeed, effective in ameliorating glomerular sclerosis. However, in some studies, glomerular sclerosis was decreased without altering glomerular hyperfiltration [9], and glomerular sclerosis occurred in some settings even in the absence of intervening hyperperfusion [10].

Thus, focus was shifted to glomerular hypertension as a key mediator of progressive sclerosis. Maneuvers that increase glomerular capillary pressure, such as therapy with erythropoietin, glucocorticoids, or high-protein diet, accelerated glomerulosclerosis, while decreasing glomerular pressure ameliorated sclerosis. These beneficial effects were particularly apparent in the comparison of agents such as ACEIs that preferentially decrease glomerular pressure even more than systemic BP to non-specific antihypertensive agents [11].

### Renin–angiotensin–aldosterone system

The RAAS has been the focus of investigation of progression in CKD because of the efficacy of inhibition of its components in CKD. ACEIs decrease glomerular capillary pressure by preferential dilation of the efferent arteriole [1], likely mediated by both inhibition of angiotensin II (AngII) and especially by the effect of ACEIs in augmenting bradykinin, which is degraded by angiotensin I converting enzyme (ACE) [12]. Indeed, angiotensin type 1 receptor blockers (ARBs), which do not have this activity to increase bradykinin, do not preferentially dilate the efferent arteriole or decrease glomerular pressures to the extent of that seen with ACEIs in most experimental studies. However, both ACEIs and ARBs have shown superior efficacy in slowing progressive CKD in experimental models and in human CKD [13–16].

ARBs leave the angiotensin type 2 (AT2) receptor active, and may in theory even lead to augmented AT2 effects by allowing unbound AngII to bind to this receptor. The AT2 receptor counteracts some of the classic AT1 receptor actions and thus is mildly vasodilating and mediates growth inhibition and apoptosis [17–20]. Apoptosis often is associated with decreased injury, as injured cells are quickly removed without activation of profibrotic cytokines and chemokines. Absence of AT2 receptor actions, either by pharmacological inhibition or by genetic absence, indeed resulted in diminished apoptosis after injury, associated with increased fibrosis [21, 22].

Combined ACEI and AT1 receptor antagonist treatment could have a theoretic advantage, allowing further blockade of AngII actions while maintaining preferential local availability of the AT2 receptor [23]. In an experimental model, combined ACEI and ARB therapy did not result in added benefit on glomerulosclerosis when compared with single-drug therapy with similar blood pressure control [24, 25]. However, addition of AT2 receptor inhibition to ARB treatment prevented the beneficial effects of ARBs [26]. A beneficial effect of the AT2 receptor in renal injury was also demonstrated in transgenic mice overexpressing the AT2 receptor. These mice developed less severe injury than did the wild type after subtotal nephrectomy [27]. Results from small clinical studies of human CKD suggest that the combination of ARBs and ACEIs has greater effect in the decrease of proteinuria, not attributable to effects on systemic blood pressure [28, 29]. In a large study of hypertensive patients with diabetic nephropathy and microalbuminuria, combined therapy resulted in greater reduction of blood pressure and albuminuria than did therapy with either drug alone [30]. In a Japanese study, in addition to decreased proteinuria, the slope of decline of glomerular filtration rate (GFR) improved with combination ACEI and ARB versus monotherapy [31]. However, complete dose-range comparisons of combined therapy with monotherapy were not made in these clinical trials. A recent review of clinical trials with combination therapy with ACEI and ARB in CKD patients support that such combination therapy had increased effects to decrease proteinuria without significantly increasing adverse side effects [16].

Antifibrotic effects of combination therapy versus monotherapy could include augmented bradykinin and AT2 activity and also decreased urinary transforming growth factor (TGF)-β [32]. In addition, there may be greater suppression of the renin–angiotensin system (RAS) with combined therapy, decreasing both ligand generation by inhibition of ACE and binding of any remaining AngII to the AT1 receptor. However, even suprapharmacological doses of ACE inhibition did not achieve complete suppression of the local RAS in experimental models [33]. Similarly, patients receiving ACEIs long term still have measurable ACE in their plasma. These data support the notion that non-ACE-dependent AngII generation by chymotrypsin-sensitive generating enzyme occurs in humans. New directions under investigation include the development of renin antagonists that could obviate these obstacles to optimal inhibition of the RAAS. Renin itself may have direct effects, independent of activation of the RAAS, with renin receptor activity detected on mesangial cells [34].

Many profibrotic actions of the RAAS are mediated directly by AngII. AngII promotes migration of endothelial and vascular smooth muscle cells, and hypertrophy and hyperplasia of smooth muscle cells and mesangial cells [35, 36]. All components of the RAS are present in macrophages, which may thus serve as yet another source of AngII and also respond to ACEI and ARB. AngII also induces other growth factors, including basic fibroblast growth factor (basic FGF), platelet-derived growth factor (PDGF) and TGF-β, and plasminogen activator inhibitor-1 (PAI-1), all of which may impact on fibrosis (see below), [37–39].

Importantly, new data indicate that aldosterone has both genomic and non-genomic actions to promote fibrosis, independent of its actions to increase blood pressure by mediating salt retention [40, 41]. Aldosterone enhances angiotensin induction of PAI-1 (see below), and also has direct actions on fibrosis [40]. Conversely, aldosterone receptor antagonism with spironolactone decreased injury [40]. PAI-1 deficiency prevented aldosterone-induced glomerular injury, but interestingly did not alter cardiac or aortic injury in this mouse model, suggesting site-specific and perhaps species-specific mechanisms of aldosterone-PAI-1 mediated fibrosis [42]. In clinical trials, aldosterone antagonism has further decreased proteinuria when added to ACEI and ARB therapy [43, 44]. However, the potential risk of hyperkalemia may limit the ability to add aldosterone antagonism to angiotensin inhibition. Whether these approaches also apply to children with CKD has not been investigated.

Clearly, the RAAS has many non-hemodynamic actions and thus, doses beyond usual antihypertensive doses are potentially of additional benefit. Regression has even been achieved in experimental models with high-dose ACEI/ARB. A shift in the balance of synthesis/degradation of extracellular matrix (ECM) must occur to accomplish regression of sclerosis; endothelial cells must regenerate, mesangial cells must regrow, and finally, podocytes must be restored. New glomeruli cannot be generated after term birth in humans. However, remaining segments of non-sclerotic loops can give rise to more open capillary area by lengthening or branching of the remaining capillaries [45–48]. Recent experimental data show that regression can, indeed, be induced by high-dose ACEI or ARB or spironolactone, linked to decreased PAI-1, restored plasmin activity and capillary remodeling [25, 49–51]. Of note, regression was not associated with increased expression or activity of matrix metalloproteases-2 or -9 or decreased mRNA for TGF-β or local decreases in TGF-β expression as assessed by in situ hybridization. However, lack of changes in mRNA does not rule out that local changes in TGF-β actions could occur, and clearly, in many systems, TGF-β has been shown to impact on ECM accumulation. Regression is also possible in human CKD, demonstrated in principle by regression of early diabetic sclerosis and tubulointerstitial fibrosis in patients over a 10-year period when the underlying diabetes was cured by pancreas transplantation [52]. Regression of existing lesions also occurred in IgA nephropathy in response to high-dose corticosteroids and tonsillectomy [53].

### Specific cytokines/growth factors and progression of CKD

Numerous cytokines/growth factors appear to modulate progression of glomerular and tubulointerstitial scarring. These factors and their roles may differ at the various stages of injury. Altered gene expressions and/or pharmacologic manipulations in pathophysiological settings have implicated e.g. PDGF, TGF-β, AngII, basic FGF, endothelin, various chemokines, peroxisome proliferator-activated receptor-γ (PPAR-γ) and PAI-1, among others, in progressive renal scarring [10, 54–56]. Current state-of-the-art approaches with proteomic and array analysis of renal tissue in human CKD and in animal models can identify novel targets and markers, and even mediators of progression [57, 58]. Of these many potential molecules of interest, we will discuss only a few that have been investigated in depth.

Increased PAI-1 is associated with increased cardiovascular disease and fibrotic kidney disease [59]. Conversely, PAI-1 could be decreased by inhibition of AngII and/or aldosterone, and linked to prevention of sclerosis or even regression of existing kidney fibrosis [25, 38, 51, 60]. AngII and aldosterone can also induce PAI-1 expression and subsequent fibrosis independent of TGF-β activation [61]. Some of the effects of PAI-1 in promoting fibrosis are independent of its effects on proteolysis. PAI-1 also modulates cell migration, perhaps by its effects on vitronectin interaction [59]. Thus, PAI-1 may in some inflammatory or interstitial disease settings increase fibrosis primarily by enhancing cell migration and epithelial-mesenchymal transition (EMT). In contrast, in the glomerulus, the effects of PAI-1 in the increase of sclerosis may predominantly be due to its ability to modulate ECM turnover [59]. These data support that mechanisms of fibrosis in the interstitium and glomerulus are not identical, and involve complex interactions of parenchymal and infiltrating cells and cytokines, with variable net effects on ECM accumulation.

TGF-β promotes ECM synthesis and is a key promoter of fibrosis. The biological actions of TGF-β are complex and depend not only on cell state, but also on the presence of decorin and latency-associated peptide (LAP), both of which can bind and modify its activity [37]. TGF-β also induces both PAI-1 and AngII [62]. Animals transgenic for TGF-β developed progressive renal disease [63]. Conversely, inhibition of either TGF-β or PDGF-B decreased mesangial matrix expansion in the anti-Thy1 model [64, 65]. Animals genetically deficient for TGF-β develop lymphoproliferative disease, thought to reflect a loss of TGF-β immune regulatory effect [66]. Interestingly, pharmacologic inhibition of TGF-β was more effective at lower dose, and with higher dose of anti-TGF-β associated with more fibrosis and greater macrophage influx, perhaps also reflecting effects on TGF-β immune modulation [67]. TGF-β may promote a more fibroblastic phenotype of the podocyte, with loss of differentiation markers and de novo expression of alpha-smooth muscle actin [68]. Although TGF-β promotes growth arrest and differentiation of podocytes at low doses, at higher doses, TGF-β causes podocyte apoptosis, mediated by Smad 7 signaling [69, 70]. Loss of podocytes (see below) is a key factor contributing to progressive kidney fibrosis.

PPAR-γ modifies numerous cytokines and growth factors, including PAI-1 and TGF-β. PPAR-γ is a transcription factor and a member of the steroid superfamily [71]. On activation, PPAR-γ binds the retinoic acid X receptor, translocates to the nucleus and binds to peroxisome proliferator activator response elements (PPREs) in selected target genes, modifying their expression. PPAR-γ agonists, such as the thiazolidinediones, are most commonly used to treat type 2 diabetes, due to their beneficial effects to increase insulin sensitivity and improve lipid metabolism, and they have been shown to decrease diabetic injury correspondingly in diabetic animal models [72]. Interestingly, PPAR-γ agonists also have antifibrotic effects in non-diabetic or non-hyperlipidemic experimental models of CKD. PPAR-γ agonist ameliorated the development of sclerosis in these non-diabetic models, linked to decreased PAI-1 and TGF-β and decreased infiltrating macrophages and protection of podocytes against injury [56, 73]. Further study is necessary to determine the specific role each of the above factors plays at varying stages of renal fibrosis.

### Podocyte loss

Podocytes are the primary target in many glomerular diseases, including FSGS and the experimental models of adriamycin and puromycin aminonucleoside-induced nephropathies [74]. The podocytes are pivotal for maintenance of normal permselectivity, and are a source of matrix in both physiological and pathophysiological settings. The podocyte does not normally proliferate. Loss of podocytes after injury is postulated to be a key factor resulting in progressive sclerosis [74]. This principle was proven in experimental models in mice and rats, where podocyte-specific injury was produced by genetic manipulation of the podocytes to express toxin receptors only on this cell [75, 76]. Injection of toxin then resulted in podocyte loss, the degree of which depended on toxin dose. Animals subsequently developed progressive sclerosis. Of interest, even though only podocytes were initially injured, subsequent injury rapidly also developed in endothelial and mesangial cells, with resulting sclerosis. Even when chimeric mice were genetically engineered so that only a portion of their podocytes was susceptible to the toxin, all podocytes developed injury after toxin exposure [77]. These data show that injury can also spread from the initially injured podocyte to initially intact podocytes within a glomerulus, setting up a vicious cycle of progressive injury at the glomerular level [77].

The limited proliferation in the mature podocyte is accompanied by high expression of a cyclin-dependent kinase inhibitor, p27kip1, a rate-limiting step for the growth response of the podocyte [78]. Either too much or too little proliferation of the podocyte in response to genetic manipulation of p27kip1 is postulated be detrimental [79]. Inadequate growth of the podocyte is postulated to give rise to areas of dehiscence and insudation of plasma proteins, which progress to adhesions and sclerosis [80]. Another cyclin-dependent kinase inhibitor, p21, appears to be necessary for development of injury after five-sixths nephrectomy in mice, pointing to the crucial importance of cell growth responses in determining response to injury [81].

Podocytes normally produce an endogenous heparin-like substance, which inhibits mesangial cell growth; thus, injury may decrease this growth inhibitory effect and allow increased mesangial growth. Podocytes are also the main renal source of angiopoietin-1 and vascular endothelial growth factor (VEGF), an endothelial cell-specific mitogen that plays a key role in both physiologic and pathologic angiogenesis and vascular permeability [82]. Overexpression or partial loss of podocyte VEGF results in a collapsing lesion or pre-eclampsia-like endotheliosis lesion, respectively [82].

### Podocyte genes and CKD

New studies of the molecular biology of the podocyte and identification of genes mutated in rare familial forms of FSGS and nephrotic syndrome, such as nephrin, WT-1, transient receptor potential cation channel-6 (TRPC-6), phospholipase C epsilon, α-actinin-4 and podocin, have given important new insights into mechanisms of progressive glomerulosclerosis. The gene mutated for congenital nephrotic syndrome, nephrin (NPHS1) is localized to the slit diaphragm of the podocyte and is tightly associated with CD2-associated protein (CD2AP) [83]. Nephrin functions as a zona occludens-type junction protein, and together with CD2AP, provides a crucial role in receptor patterning and cytoskeletal polarity and also provides signaling function of the slit diaphragm [84]. Mice with CD2AP knockout develop congenital nephrotic syndrome, similar to congenital nephrotic syndrome of Finnish type [85]. Autosomal dominant FSGS with adult onset is caused by mutation in α-actinin 4 (ACTN4) [86]. This is hypothesized to cause altered actin–cytoskeleton interaction, causing FSGS through a gain-of-function mechanism, in contrast to the loss-of-function mechanism implicated for disease caused by the nephrin mutation [85]. Patients with α-actinin 4 mutation progress to end-stage by age 30 years, with rare recurrence in a transplant. TRPC-6 encodes for a cation channel, which is present in several sites including podocytes. TRPC-6 is mutated in some kindreds with familial FSGS with adult onset in an autosomal dominant pattern [87]. Podocin, another podocyte-specific gene (NPHS2), is mutated in autosomal recessive FSGS with childhood onset with rapid progression to end-stage kidney disease [88]. Podocin interacts with the CD2AP-nephrin complex, indicating that podocin could serve in the structural organization of the slit diaphragm. In some series of steroid-resistant pediatric patients with non-familial forms of FSGS, a surprisingly high proportion, up to 25%, had podocin mutations [89, 90]. However, not all patients with nephrotic syndrome caused by mutation are steroid resistant. Diffuse mesangial sclerosis in a large kindred was recently linked to a truncating mutation of phospholipase C epsilon (PLCE1), and two of those patients responded to steroid therapy [91]. However, in two patients with missense mutation of this same gene, FSGS lesions developed, demonstrating that a spectrum of structural abnormalities may arise from varying mutations in the same gene. PLCE1 is expressed in the glomerulus, where it is postulated to play a key role in development, perhaps by interacting with other proteins that are crucial for the development and function of the slit diaphragm.

WT-1 mutation, which may occur sporadically with only FSGS or be associated with Denys–Drash syndrome, was found in only 5% of steroid-resistant patients [92]. Interestingly, mutations of podocin or WT-1 were not found in relapsing or steroid-dependent pediatric patients [93]. Acquired disruption or polymorphisms of some of these complexly interacting molecules have been demonstrated in experimental models and in human proteinuric diseases. Thus, in puromycin aminonucleoside nephropathy, a model of FSGS, nephrin localization and organization were altered [94]. Similar decreases in nephrin were observed in hypertensive diabetic rat models with significant proteinuria [95]. TRPC-6, a calcium channel, was induced in various non-genetic human proteinuric diseases [96]. Conversely, treatments that ameliorated these experimental models preserved e.g. glomerular nephrin expression, providing further support for a key causal role for slit diaphragm and key podocyte molecules in proteinuria [97]. Whether polymorphisms, compound heterozygosity for mutations or merely altered distribution and/or expression of any of these proteins contribute to proteinuria or progressive disease in various causes of CKD in humans has not been determined.

### Dyslipidemia

Patients with CKD frequently have dyslipidemia and greatly increased cardiovascular disease risk, even beyond that predicted by lipid abnormalities [98]. Abnormal lipids are important in modulating glomerular sclerosis in rats; however, analogous studies in humans are still evolving [99–102]. Glomerular injury was increased in experimental CKD when excess cholesterol was added to the diets. Glomerular disease has been reported in the rare familial disease, lecithin cholesterol acyltransferase deficiency, and with excess apolipoprotein E. However, renal disease is not typical in the more common forms of primary hyperlipidemias. Patients with minimal-change disease or membranous glomerulonephritis, characterized by hyperlipidemia as part of their nephrotic syndrome, usually do not develop glomerular scarring. However, recently, post hoc and meta-analyses of clinical trial data support that abnormal lipids are associated with increased loss of GFR and that treatment with statins may not only benefit cardiovascular disease risk, but also be of benefit for progressive CKD. A post hoc analysis suggests that statins may even slow progression in patients with stage 3 CKD [102]. These beneficial effects of statins appear to extend beyond their lipid-lowering effects [98, 101].

### Proteinuria

Proteinuria is a marker of renal injury, reflecting loss of normal permselecitvity. Further, proteinuria itself has been proposed to contribute to progressive renal injury inflammation [74, 103]. Increased proteinuria is associated with worse prognosis [104]. Whether proteinuria is merely a marker of injury or a contributor to progressive injury has been debated.

Albumin can in vitro in tubular cells increase AngII and in turn upregulate TGF-β receptor expression [105]. However, in most settings, pure albumin per se is not directly injurious. Other filtered components of the urine in proteinuric states, such as oxidized proteins, appear to be more potent in inducing direct injury of tubular epithelial cells and activating proinflammatory and fibrotic chemokines and cytokines. Complement and various lipoproteins are also present in the urine in proteinuric disease states and can activate reactive oxygen species [101, 106]. Proteinuria may thus alter tubule cell function directly, potentially contributing to a more profibrotic phenotype, and also augment interstitial inflammation, in particular by macrophages. Proteinuria may activate many profibrotic pathways through its ability to increase NF-kB, and also by other pathways. These include for instance complement synthesis from tubules [107].

Interventions that are particularly effective in decreasing proteinuria, such as the administration of ACEIs or ARBs, also decrease overall end organ injury. Whether these beneficial effects are dependent on the reduction of proteinuria has not been proven, in that these interventions have multiple parallel effects that may all contribute to the decrease of fibrosis [107].

### Mechanisms of tubulointerstitial fibrosis

Tubulointerstitial fibrosis classically was thought merely to reflect glomerular injury and resulting whole nephron ischemia in most CKD. Interesting new data point to independent mechanisms of interstitial fibrosis and the importance of the tubulointerstitial lesion in progression. Decreased peritubular capillary density, possibly modulated by decreased VEGF or other angiogenic factors, has been proposed as a mechanism in various progressive renal diseases [108]. Future studies may demonstrate whether these interstitial microvascular lesions are causal or consequential in the development of interstitial injury.

Increased numbers of macrophages are closely correlated with both glomerulosclerosis and tubulointerstitial fibrosis and are usually decreased by interventions that decrease fibrosis. These cells are potential sources of numerous cytokines and eicosanoids that affect the glomerulus [109]. Support for this hypothesis is seen with the protective effects of maneuvers that decrease macrophage influx. In a rat model of unilateral ureteral obstruction (UUO), administration of ACEI ameliorated interstitial monocyte/macrophage infiltration and decreased fibrosis [110]. Studies in β6 integrin-deficient mice revealed that infiltrating macrophages do not inevitably transduce fibrotic effects; in these mice local activation of TGF-β is impaired, and they are protected from fibrosis despite abundant macrophage infiltration [61]. Macrophages may even play a beneficial role in scarring. The specific role of the macrophage AT1a receptor in renal fibrosis was examined in studies of bone marrow transplantation in wild type mice with UUO mice reconstituted with either wild type macrophages or macrophages devoid of the AT1a receptor. There was more severe interstitial fibrosis in mice with the AT1a deficient macrophages, even though fewer infiltrating macrophages were observed, suggesting that the macrophage AT1a receptor functions to protect the kidney from fibrogenesis [111].

In human diabetic nephropathy there is an early increase in total interstitial cell volume (which may represent increased cell size and/or number), preceding the accumulation of interstitial collagen [112]. This is in contrast to the diabetic glomerular lesion, where the expanded mesangial area is largely due to increased matrix accumulation rather than hypercellularity. These interstitial cells could possibly represent interstitial myofibroblasts, postulated to play a key role in interstitial fibrosis. These activated interstitial cells are a major source of collagen synthesis, and increased expression of α-smooth muscle actin (SMA), a marker of myofibroblasts, predicts progressive renal dysfunction both in human and experimental renal disease.

The source of interstitial myofibroblasts is a topic of controversy. Bone marrow-derived or potential renal stem cells may give rise not only to interstitial cells but also to regenerating parenchymal cells [113]. Epithelial–mesenchymal transformation (EMT) is another possible mechanism for generation of interstitial myofibroblasts [114]. This seamless plasticity of cells changing from epithelial to mesenchymal phenotypes exists during early development. EMT may also occur in the adult after injury, contributing approximately half of the interstitial fibroblasts in experimental models [114]. Injured tubular epithelial cells can change phenotype both in vivo and in vitro, with de novo expression of a fibroblast-specific protein (FSP1), and possibly migrate into the interstitium as myofibroblasts. The surrounding matrix and basement membrane underlying the tubular epithelium is disrupted by local proteolysis, modulated by an array of cytokines and growth factors, including insulin-like growth factors I and II, integrin-linked kinases, EGF, FGF-2 and TGF-β [114]. Several key factors inhibit EMT, including hepatocyte growth factor and bone morphogenetic factor-7, and thus inhibit fibrosis in experimental CKD [114].

### Anatomic and genetic risks for CKD: nephron number and gene polymorphisms

Risk for development of CKD and its rate of progression varies in differing populations. CKD associated with hypertension and arterio-nephrosclerosis is particularly common in African Americans, and FSGS is more frequently the underlying cause of steroid-resistant FSGS in African Americans and Hispanics than in Caucasians [115, 116]. These varying disease trends in differing ethnic populations could represent both genetic and environmental factors. Low birth weight is epidemiologically linked to increased risk for cardiovascular disease, hypertension and CKD in adulthood. The link is postulated to be due to the decreased nephron number that accompanies low term birth weight, defined as less than 2,500 g [117, 118]. These fewer nephrons are postulated to be under greater hemodynamic stress, thus contributing to progressive sclerosis. Of interest, low birth weight is much more common in African Americans than in Caucasians and is not accounted for by socioeconomic status [119]. Further, glomerular size in normal African Americans is larger than in Caucasians and could possibly reflect smaller nephron number [120]. In Australian Aborigines, marked increase in incidence of CKD is associated with larger but fewer glomeruli and low birth weight [121, 122]. Mechanisms other than hemodynamic stress that could underlie these differences in normal glomerular populations and also relate to increased incidence of end-stage renal disease include functional polymorphisms of genes that are involved both in renal/glomerular development and contribute to amplified scarring mechanisms, such as the renin–angiotensin system [10].

African Americans also have increased severity of renal disease associated with several systemic conditions. The course of lupus nephritis in a prospective trial was more severe in African Americans than in Caucasians, with more extensive crescent formation and interstitial fibrosis and greater likelihood of end-stage renal disease [123]. Even the manifestations of HIV infection in the kidney differ markedly between African Americans and Caucasians: HIV-associated renal disease in African Americans is typically an aggressive collapsing type of FSGS, contrasting lower grade immune-complex-mediated glomerulonephritides in Caucasians with HIV infection and renal disease [124]. Genetic background also modulates susceptibility in experimental models, both to podocyte injury (e.g. only the balb/c mouse strain is susceptible to adriamycin) and to hypertension injury (e.g. in the five-sixths nephrectomy model, C57Bl mice are resistant, 129Sv/J mice are susceptible) and even to diabetic injury [125–127].

There is also accumulating evidence that specific genes in humans modulate the course and rate of organ damage. Polymorphisms in several genes within the RAAS system, including ACE, angiotensinogen and the angiotensin type 1 receptor, have been linked with cardiovascular and renal disorders, including diabetic nephropathy, IgA nephropathy and uropathies [128–133]. The ACE DD genotype, associated with increased RAS activity, was increased in patients with IgA nephropathy who ultimately experienced progressive decline in renal function during follow-up compared with those whose function remained stable over the same time [134].

Polymorphisms of TGF-β are also implicated in hypertension and progressive fibrosis. The Arg 25 polymorphism may be increased in African Americans, who may also have greater elevation of circulating TGF-β when they reach end-stage renal disease than do Caucasians [135].

These observations suggest that complex genetic traits can modulate the response of glomerular cells to pathogenic stimuli in experimental models. Whether ethnic differences in development of renal disease in humans reflect contributions of genetic and/or environmental influences remains to be definitively determined.

QUESTIONS (Answers appear following the reference list)

1. A 6-year-old African American boy presented with generalized edema, 24 h urine protein excretion of 1.5 g and normal complements, and serum creatinine of 0.7 mg/dl. His blood pressure was 110/70 mmHg. His nephrotic syndrome did not respond to an 8-week course of steroids, and a renal biopsy is planned. The most likely diagnosis in this patient is:
  - FSGS due to mutation of podocin
  - Minimal-change disease
  - FSGS, usual type
  - Diffuse mesangial sclerosis
  - Collapsing glomerulopathy
2. For the same patient detailed in question 1, what additional treatment should be initiated at this time to decrease risk of CKD:
  - Diuretics
  - Spironolactone
  - ACEIs
  - Beta blockers
  - ACEIs and ARBs
3. In the same patient detailed in the above questions, what parameters would be most important to follow and evaluate for adjustment of therapy:
  - Edema
  - White blood cell (WBC) count
  - Blood pressure
  - Proteinuria
4. A 14-year-old Caucasian girl was diagnosed with IgA nephropathy, which on biopsy showed fibrocellular crescents, with focal proliferative and secondary sclerosing lesions of glomeruli. Her urine protein excretion was 1.0 g in 24 h. Urinalysis showed frequent red blood cell casts, serum creatinine was 1.2 mg/dl and her blood pressure was 120/93 mmHg. Which of the following mechanisms are likely to contribute to progression of her CKD:
  - Podocyte loss
  - Proteinuria
  - Glomerular hypertension
  - Infiltrating macrophages
  - All of the above
5. A 10-year-old Caucasian boy with a history of multiple episodes of steroid-dependent nephrotic syndrome since the age of 4 years now has proteinuria of 3.8 g in 24 h, with unremarkable urinalysis without red blood cell casts; his serum creatinine is 0.6 mg/dl, and his blood pressure is 98/64 mmHg. He has an increased cholesterol level of 480 mg/dl and triglyceride levels are 110 mg/dl. What mechanisms of renal injury are likely to be activated in this child:
  - podocyte loss
  - proteinuria
  - dyslipidemia
  - glomerular hypertension
  - (b) and (c)
